# Unraveling Early Onset Disparities and Determinants: An Analysis of Colorectal Cancer Outcomes and Trends in Texas

**DOI:** 10.7759/cureus.83124

**Published:** 2025-04-28

**Authors:** Tyler Torres, Elias Arellano Villanueva, Yossef Alsabawi, Demba Fofana, Manish K Tripathi

**Affiliations:** 1 General Surgery, University of Texas Rio Grande Valley School of Medicine, Edinburg, USA; 2 Internal Medicine, University of Texas Rio Grande Valley School of Medicine, Edinburg, USA; 3 Otolaryngology, University of Texas Rio Grande Valley School of Medicine, Ediburg, USA; 4 Mathematics and Statistics, University of North Carolina Greensboro, Greensboro, USA; 5 Cancer and Immunology, Research Faculty, University of Texas Rio Grande Valley, Edinburg, USA

**Keywords:** colorectal cancer, early-onset crc, health disparities, racial/ethnic differences, survival disparities, texas cancer registry

## Abstract

Introduction

Colorectal cancer (CRC) is the second leading cause of cancer-related deaths in the U.S., with disparities in incidence, survival, and age at diagnosis across racial, ethnic, and socioeconomic groups. The rising incidence of early-onset CRC (<50 years) has amplified concerns regarding access to care, screening disparities, and outcomes, particularly among minorities. This study examines the impact of race, ethnicity, socioeconomic status (SES), and sex on CRC survival and age at diagnosis in Texas from 1995 to 2016.

Methods

This retrospective cohort study utilized Texas Cancer Registry (TCR) data, including 235,076 CRC cases diagnosed between 1995 and 2016. Kaplan-Meier analysis and log-rank tests assessed 10-year survival by race and ethnicity and time period (1995-2005 vs. 2006-2016). Kruskal-Wallis tests with Bonferroni correction were used to compare survival years between racial/ethnic groups within each period. Age at diagnosis was analyzed by race and ethnicity, and SES using Welch ANOVA and Games-Howell post hoc testing. Welch’s t-tests compared intra-race changes across decades. Sex-based differences in diagnosis age were assessed using Mann-Whitney U tests.

Results

Significant racial, ethnic, and socioeconomic disparities were observed in CRC outcomes. Black: Non-Hispanic and Black: Hispanic patients exhibited some of the lowest median survival times, with minimal overall improvement between the two time periods. Although Black: Hispanic patients exhibited the lowest median survival, the difference was not statistically significant in the 2006-2016 cohort (p = 0.12). Hispanic and Black patients were diagnosed at younger ages compared to White: Non-Hispanic patients. Lower SES was associated with younger age at diagnosis and worse survival. Male patients were consistently diagnosed earlier than female patients across both decades. Despite some improvement in survival for certain groups, disparities persisted, particularly for Black: Non-Hispanic and Black: Hispanic patients.

Conclusion

Disparities in CRC survival and diagnosis age persist across racial, ethnic, SES, and sex lines in Texas. These findings underscore the need for tailored screening efforts, improved healthcare access, and targeted interventions for high-risk populations. Persistent sex-based differences highlight a need for further research into biological and systemic factors. Addressing social determinants of health may help reduce these disparities.

## Introduction

Colon and rectal, or colorectal cancer (CRC), is classified as the second leading cause of cancer-related deaths in the United States in 2023 [1]. According to the American Cancer Society, in 2025, approximately 154,000 individuals are expected to be diagnosed with CRC, with over 52,000 patients expected to die from the disease in the United States [2]. Within the state of Texas, in 2022, there were an estimated 12,444 Texans diagnosed with CRC and nearly 4,500 deaths (Texas Department of Health Services). Between 1995 and 2020, the incidence rate of CRC was 39 new cases for every 100,000 Texans [3]. 

Like many aspects of healthcare, survival rates of CRC demonstrate sex, geographic, and ethnic variation. According to UK and US data, the overall incidence of CRC is higher in males than females, with the incidence rate ratio being 1.38 in the retrospective US study [4,5]. According to White et al., this disparity is likely influenced by a combination of biological, genetic, and behavioral factors, with females more commonly associated with genetic mutations. CRC outcomes have also been shown to be affected by geographic factors. Studies in Europe have shown that Eastern Europeans have lower survival rates than other Europeans, emphasizing that geographic factors affect mortality and survival in patients with CRC [6]. These variations may hold true for other geographical distributions as well.

In the United States, regional disparities in CRC outcomes are often closely tied to the racial and ethnic composition of local populations. In Texas, this is particularly evident along the Texas-Mexico border, where counties with high proportions of Hispanic residents exhibit significantly lower CRC screening rates, some as low as 34%, compared to the national average of 71.8% [7-9]. While these regions show a lower reported CRC incidence, this is believed to reflect underdiagnosis rather than a lower disease burden due to reduced access to care and screening infrastructure [7]. Wang et al. reported a rising CRC incidence in Texas' young Hispanic population, with no corresponding decline among screening-age Hispanics [10]. National data show similar trends: Hispanic patients have persistently lower screening rates, higher mortality, and more frequent late-stage diagnoses than Non-Hispanic patients [11,12]. These findings support that CRC disparities are not simply geographic; they are driven by structural inequities disproportionately affecting racial and ethnic minorities, particularly Hispanic populations in underserved areas. Thus, regional variation in CRC outcomes serves as a proxy for deeper racial and ethnic disparities that merit stratified investigation.

Other studies have shown that regional differences in CRC exist because of other factors contributing to the disease process, including socioeconomic status (SES) and poverty index. SES is a critical social determinant of health, significantly influencing outcomes across nearly all areas of healthcare. According to Beckmann et al., CRC patients from the most socioeconomically advantaged areas had significantly better outcomes than those from the least advantaged areas [13]. This is consistent with American literature, where SES was found to be an independent predictor of CRC stage at diagnosis, with cases from the highest SES group more likely to present with local stage disease than those from the lowest SES group [14]. A study conducted in New Jersey found that geographical differences in mortality were greatly influenced by the patient's surrounding community. It was observed that Black patients and people living in underserved areas may be at greater risk of poor outcomes compared with White patients and persons living in wealthy areas [15]. Similarly, a recent analysis of CRC diagnosis has shown that individuals with a median income of less than USD 55,000 a year are 21% more likely to be diagnosed with distant CRC compared to wealthier counterparts [12]. Thus, ethnicity and SES are contributing factors that may help explain the survival disparities in CRC mortality within a specific region. 

Another significant factor contributing to the mortality of CRC in Texas is the age at diagnosis. Incidence rates of early-onset CRC (<50 years of age) have increased in the United States in the past two decades, contrasting the steady decline in the incidence of later-onset CRC [16]. This rise in early-onset CRC in the United States is concerning, given that younger patients with CRC often experience delayed diagnosis as they may not adhere to regular screening guidelines, which typically begin at 50 [17]. Furthermore, younger CRC patients are more likely to present with an aggressive form of the disease [17].

Additionally, various risk factors, including genetic predisposition, obesity, smoking, SES, and inflammatory conditions like Crohn’s and ulcerative colitis, contribute to the rising incidence among younger populations [17]. Interestingly, ethnicity has been shown to impact prognosis despite lower incidence rates of early-onset CRC; Hispanic and Asian patients exhibited poorer survival outcomes compared to White patients. Similarly, in a study on five-year survival in patients with early-onset CRC, it was discovered that Hispanic, Black, and Asian patients had a lower relative survival rate compared with White patients [18]. 

In this study, we used data from the Texas Cancer Registry (TCR) to assess two primary outcomes: differences in survival time and age at diagnosis of CRC across racial and ethnic groups, stratified by two time periods: 1995-2005 and 2006-2016. Specifically, we aimed to determine whether survival outcomes and age at diagnosis significantly changed over time within and between racial/ethnic categories and whether race, ethnicity, sex, and SES were associated with early-onset CRC (<50 years). We placed a particular focus on Black: Hispanic and Hispanic populations based on existing literature highlighting that these groups experience lower screening rates, delayed diagnoses, and poorer survival outcomes due to structural barriers to healthcare access [10-12,18,19]. Given this evidence and the unique demographic landscape of Texas, we hypothesize that Black: Hispanic individuals will demonstrate shorter survival following a CRC diagnosis compared to other racial and ethnic groups, and that Hispanic individuals overall will have an earlier age at diagnosis compared to their Non-Hispanic counterparts.

This article was previously presented as a poster and published as an abstract at the 2024 American Association for Cancer Research (AACR) 17^th^ AACR Conference on The Science of Cancer Health Disparities in Racial/Ethnic Minorities and the Medically Underserved on September 21^st^, 2024. 

## Materials and methods

Study design

This study is a retrospective cohort study utilizing data from the TCR to examine disparities in colorectal cancer survival and age at diagnosis across racial, ethnic, socioeconomic, and sex groups. The cohort consists of 235,076 patients diagnosed with CRC between 1995 and 2016, with data accessed through SEER*Stat software (National Cancer Institute, Bethesda, MD) for analysis. Patients were categorized by race, ethnicity, SES, and sex, and their outcomes, age at diagnosis, and survival time were analyzed over two distinct time periods: 1995-2005 and 2006-2016.

Variables 

Anaconda Navigator version 2.5.0 (Anaconda, Inc., Austin, TX) and Jupyter Notebook version 6.4.5 (Project Jupiter, https://jupyter.org) running Python version 3.9 (Python Software Foundation (PSF), Beaverton, OR) were utilized for filtering and cleaning the data. Through Jupyter Notebook, functions were developed to turn the categorical variables from the dataset into integers for analysis; this was done for all the variables seen in Table 1. Sex was coded as male/female, with the female variable as the reference category (Table 1). The variables race and ethnicity were not combined; anyone who reported they were Hispanic in the Hispanic variable was coded as such in addition to their race. Anyone reporting Non-Hispanic in the Hispanic variable was coded as the race they specified in the race variable, in addition to Non-Hispanic, so our race categories were not mutually exclusive. This was done to see the primary effects of ethnicity on CRC diagnosis in certain races. Therefore, race and ethnicity were reported together to create six variables: Black: Hispanic, Black: Non-Hispanic, White: Hispanic, White: Non-Hispanic, Other: Hispanic, and Other: Non-Hispanic (Table 1). While this approach allows for a more nuanced understanding of disparities, we acknowledge the potential for overlapping population effects. Multicollinearity was considered during model development, and covariates were selected to minimize redundancy and ensure model stability in regression analyses.

**Table 1:** Descriptive Statistics of the Patient Population Demographic Characteristics of CRC Patients in Texas, 1995–2016. All Values Are Reported As Frequencies (N) and Percentages (%) Unless Otherwise Specified; SD: Standard Deviation IQR: Interquartile Range; CRC: Colorectal Cancer

Variable	Total
Survival Years	Median= 3.50
	IQR = 7.58
	µ = 5.63
	(SD=5.79)
Vital Status	n(%)
Dead	151659 (64.51%)
Alive	83417 (35.49%)
Race/Ethnicity	
White: Hispanic	47331(20%)
White: Non-Hispanic	151606 (64%)
Black: Hispanic	302 (0.12%)
Black: Non-Hispanic	29802 (13%)
Other: Hispanic	454 (0.19%)
Other: Non-Hispanic	5581 (2%)
Sex	
Male	126640 (53.8%)
Female	108436 (46.1%)
Age (Years)	
Between 0-39	7851 (3.35%)
Between 40-49	19870 (8.45%)
Between 50-59	45332 (19.28%)
Between 60-69	59326 (25.23%)
Between 70-79	57986 (24.67%)
Above 80	44711 (19.02%)
Socioeconomic Status	
Affluent	29475 (14%)
Upper-Middle Income	40425 (20%)
Lower Middle Income	66241 (33%)
High Poverty	58767 (49%)
Unknown Poverty Level	6897 (3%)

Using race and ethnicity as a combined variable in colorectal cancer research is supported by several studies that emphasize the importance of accounting for genetic and environmental factors together to understand disease risk and outcomes. For instance, Thomas et al. demonstrated that combining genome-wide association study (GWAS) data from Asian and European populations significantly improved risk prediction for CRC across diverse populations, highlighting the limitations of European-centric models when applied to non-European groups [20]. Furthermore, Seagle et al. identified significant racial and ethnic differences in germline pathogenic variants in early-onset CRC patients, emphasizing the need for multigene panel tests that are representative of diverse populations to ensure equitable clinical applications [21]. Moreover, Khor et al. found that excluding race and ethnicity from CRC recurrence risk prediction models reduced algorithmic fairness, potentially leading to inappropriate care recommendations for minoritized groups. Including these variables improved model calibration and predictive accuracy, emphasizing their necessity for inequitable decision-making [22]. Collectively, these findings underscore the rationale for combining race and ethnicity in our analysis to enhance the accuracy, representativeness, and equity of CRC risk prediction and treatment strategies. 

In this study, SES was categorized into five groups based on poverty levels, as reported by Surveillance, Epidemiology, and End Results (SEER) data: 0%-<5% poverty, 5%-<10% poverty, 10%-<20% poverty, 20%-100% poverty, and unknown poverty level [23,24]. To enhance readability, we recategorized these groups using more intuitive labels: affluent (0%-<5% poverty), upper middle-income (5%-<10% poverty), lower middle-income (10%-<20% poverty), high poverty (20%-100% poverty), and unknown SES, as shown in Table 1.

Analytical methods 

All data analyses were performed using IBM SPSS Statistics software Version 29.0.0.0 (IBM Corp., Armonk, NY). Prior to any analysis, dummy variables were created in SPSS for all the variables that were recorded in Table 1. Additionally, a new variable, "survival years", was generated by utilizing the CRC data variable “survival months” and dividing it by 12. Before assessing the association between the difference in the 10-year survival rate of Hispanic Black patients compared to other races and ethnicities' descriptive tests and tests for normality were performed to ensure proper analysis. The Kolmogorov-Smirnov test was performed on the variable "race and ethnicity" to assess normality. The test showed that the distribution of each variable within the race and ethnicity category across survival years was right-skewed and departed significantly from normality. In addition to the Kolmogorov-Smirnov test, we calculated skewness and kurtosis values for the survival months variable, which was used to derive survival years. The results indicated a moderate right skew (skewness = 1.35) and mild leptokurtosis (kurtosis = 0.71), further supporting the use of non-parametric tests. While data transformations were considered, they did not significantly improve normality or interpretability, reinforcing the robustness of our chosen approach. Given the results and the sample size, we opted for utilizing non-parametric tests for the analysis of our hypotheses. 

Moreover, for the analysis of the relationship between age at diagnosis and race and ethnicity, sex, and SES, a variety of tests on the variable age at diagnosis were performed to not violate the assumptions of the ANOVA used in the analysis. A normal population distribution, independence of observations, and no significant outliers were found via descriptive analysis of the variable age at diagnosis, as well as a normal P-P plot. While performing the ANOVA, Levene’s test for homogeneity of variances was significant (p<=.001), given this result, we opted to utilize a Welch ANOVA in SPSS for the analysis of the relationship between age at diagnosis of race and ethnicity and SES. Pairwise comparisons between race and ethnicity and SES groups were conducted using the Games-Howell post-hoc test. This method was chosen due to its robustness in handling unequal variances and sample sizes. A nonparametric Mann-Whitney U test was utilized to compare the distribution of age at diagnosis of CRC across categories of sex. This decision was made given that Levene’s test was also significant (p <= .001) and therefore suggested that the data did not meet the assumption of normality.

Hypotheses analysis 

Survival analysis was performed using the Kaplan-Meier estimator to compare the 10-year survival rates between Hispanic Black and other races/ethnicities, as well as between two time periods (1995-2005 and 2006-2016). The log-rank test was used to assess statistical significance between survival curves. To evaluate differences in survival years across race and ethnicity categories within each time period, a Kruskal-Wallis test was performed, with a Bonferroni correction applied for multiple comparisons. Additionally, a Welch ANOVA and Games-Howell post-hoc test were used to compare age at diagnosis across race and ethnicity groups and SES categories. For intra-race comparisons of age between the two time periods, a Welch T-test was utilized due to unequal variances.

Data were stratified by sex and SES using population-level data for the analysis of age at diagnosis of CRC by Sex or SES. The Mann-Whitney U test was employed to evaluate differences in the age at diagnosis of CRC between males and females for each time period (1995-2005 and 2006-2016). The test statistic (U) and standardized Z-scores were reported, along with the corresponding p-values to assess statistical significance. Mean ages for each sex were calculated and compared. SES, as stated previously, was categorized into five groups based on poverty levels: affluent, upper middle-income, lower middle-income, high poverty, and unknown poverty level, as shown in Table 1. To examine the influence of SES on the age at diagnosis, box plots were generated to visualize the distribution of ages across SES categories for both time periods (1995-2005 and 2006-2016). Mean differences between groups, 95% confidence intervals (CIs), and p-values were reported. SES groups with "unknown poverty level" were included in the analyses to account for all available data.

For all analyses, statistical significance was defined as a p-value < 0.05. Results were interpreted to highlight survival years and trends in age at diagnosis across time periods and between groups, focusing on differences by sex and SES. Standardized test statistics (Z-scores) were included for the Mann-Whitney U tests to provide normalized measures of the differences.

## Results

Descriptive statistics 

A total of 235,076 patients were included for analysis. Descriptive statistics for survival years, vital status, race/ethnicity, age, and SES are shown in Table 1. Patients were widely distributed in race, ethnicity, age, and SES. Patient ages ranged from 0 to 80+ years old, with the most common age range being 60-69 years of age (25.5%). Patients were most commonly male (n=126,640, 53.9%), White: Non-Hispanic (n=151,606, 64.5%), and White: Hispanic (n=7331, 20.1%). The patient's vital status was mostly dead (n=151,659, 64.5%). The mean survival years was 5.63 (SD = 5.79) with a median survival of 3.50 years and an interquartile range (IQR) of 7.58 years. The mean age at diagnosis for all races and ethnicities within the time periods of 1995-2005 was µ=67.98 years, SD=13.83 years, and 2006-2016 was 65.50 years, SD=14.07 years. SES data showed that patients were distributed as follows: 0%-<5% poverty (n = 29,475, 14%), 5%-<10% poverty (n = 40,425, 20%), 10%-<20% poverty (n = 66,241, 33%), 20%-100% poverty (n = 58,767, 49%), and unknown poverty level (n = 6,897, 3%). This distribution highlights the significant proportion of patients residing in higher poverty levels (20%-100% poverty), accounting for nearly half of the cohort. 

Comparison between race and ethnicity and survival time within a 10-year period from 1995-2005 and 2006-2016 

**Figure 1:**
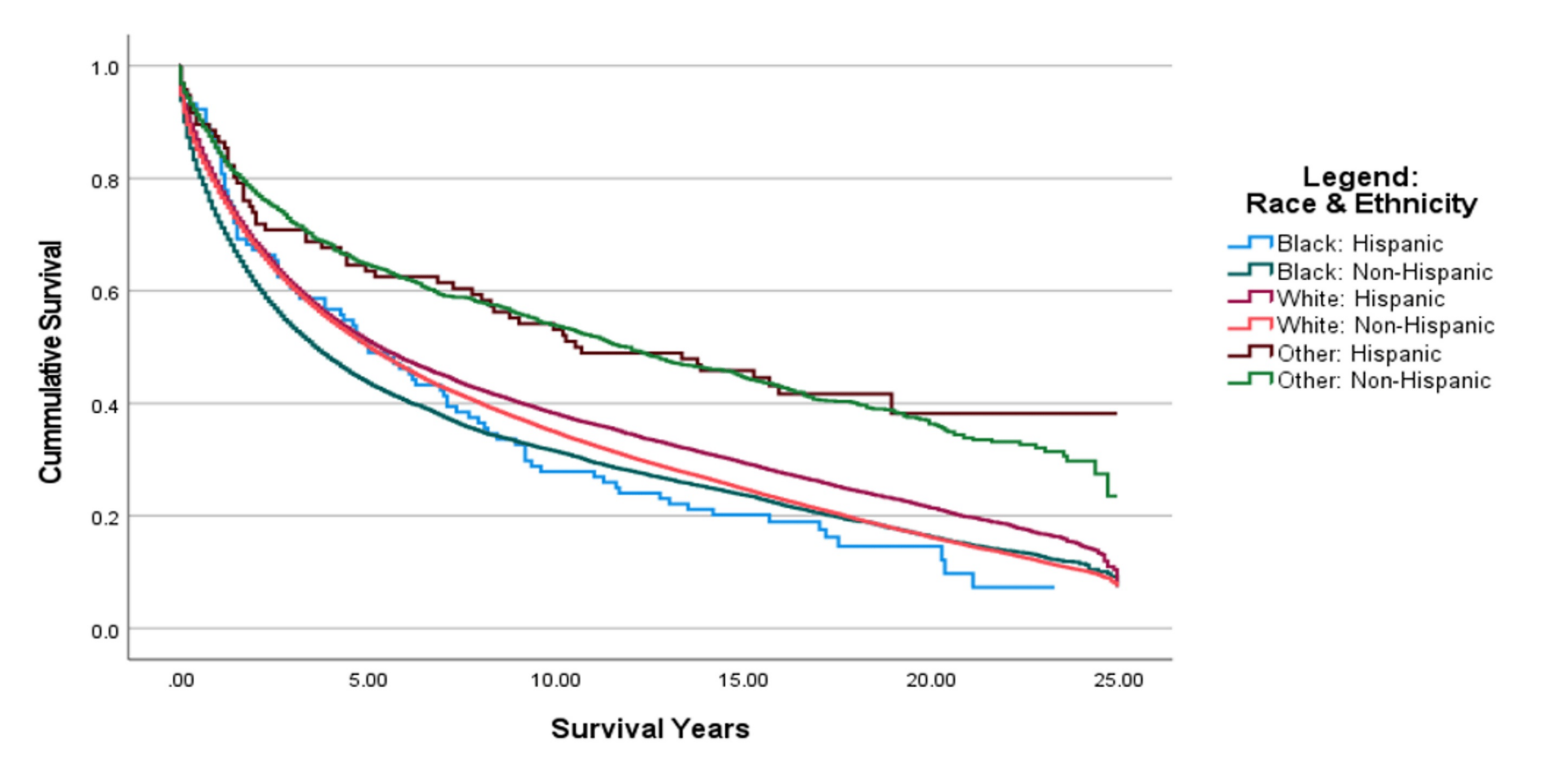
Cumulative Survival of Diagnosed CRC Patients by Race and Ethnicity From 1995–2005 CRC: Colorectal Cancer

**Figure 2:**
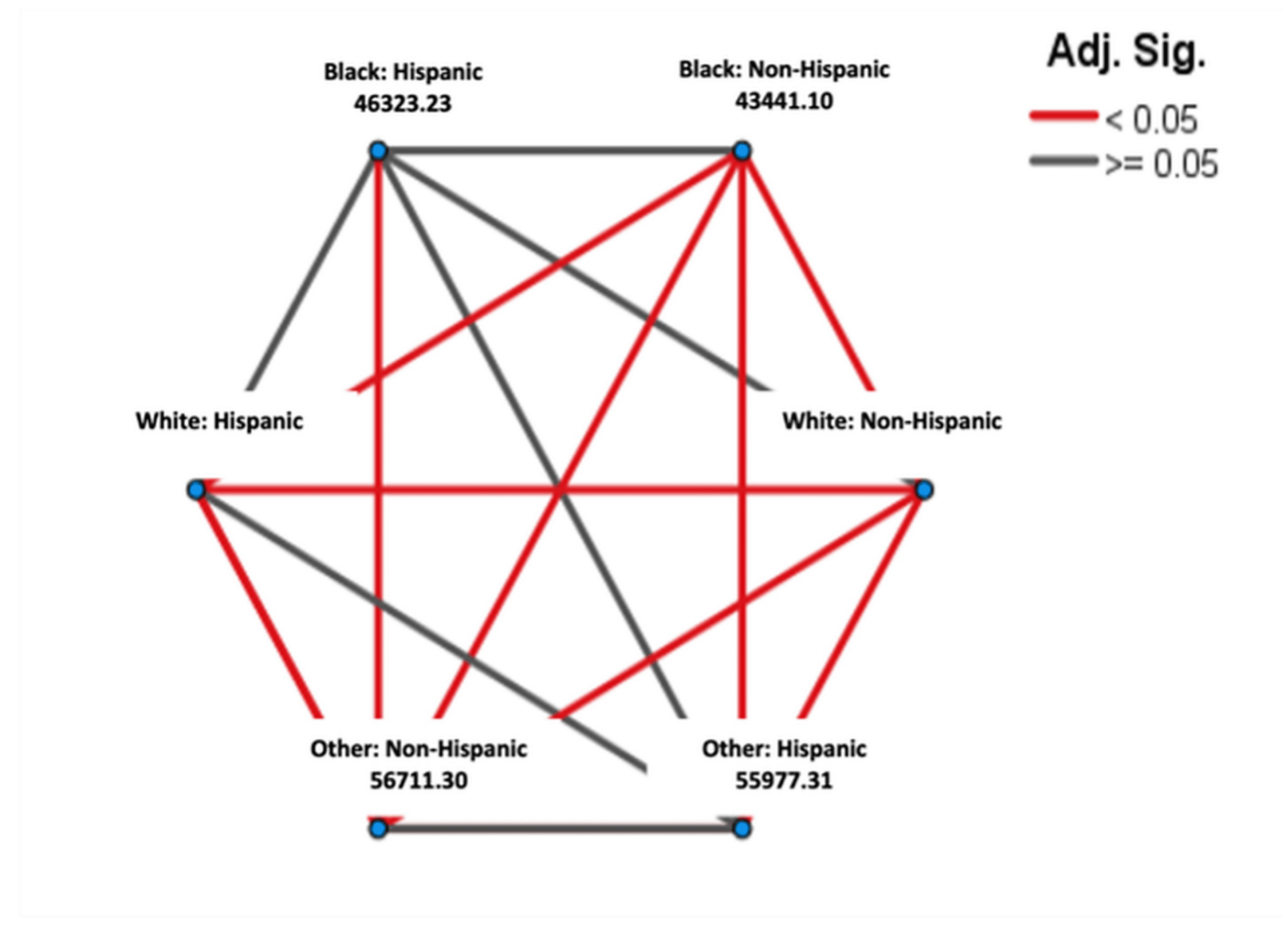
Pairwise Comparison of Survival Time Among Race/Ethnicity From 1995–2005

From 1995 to 2005, as shown in Figure 1, when comparing the Kaplan-Meier curve between all races in the 10 years, it was shown that Black: Non-Hispanic patients had the lowest median survival (estimate = 3.583 years), followed by Black: Hispanic patients (estimate = 5.0 years) and White: Non-Hispanic patients (estimate = 5.0 years). Other: Non-Hispanic patients displayed the highest survival median (estimate = 11.833 years) (Table 2). The pairwise comparison of survival time among races and ethnicities between the years 1995 and 2005 showed that the Black: Non-Hispanic population was significantly different from all other races and ethnicities except Black: Hispanic (p < .001), as shown in Figure 2. The Black: Hispanic population was only significantly different from the Other: Non-Hispanic population (p<.001), and the White: Hispanic population was significantly different from the Black: Non-Hispanic population, the White: Non-Hispanic population, and Other: Non-Hispanic population (p<.001), as shown in Figure 2. 

**Table 2:** Median Survival Time From 1995–2016 Across All Races and Ethnicities

Year of Diagnosis	Race and Ethnicity	Median	95% Confidence Interval	
			Lower Bound	Upper Bound
1995-2005	Black: Hispanic	5.00	3.40	6.60
	Black: Non-Hispanic	3.58	3.41	3.76
	White: Hispanic	5.33	5.10	5.57
	White: Non-Hispanic	5.00	4.91	5.09
	Other: Hispanic	10.50	4.39	16.61
	Other: Non-Hispanic	11.83	10.44	13.22
2006-2016	Black: Hispanic	4.25	2.67	5.83
	Black: Non-Hispanic	4.75	4.56	4.94
	White: Hispanic	6.33	6.14	6.53
	White: Non-Hispanic	5.75	5.65	5.85
	Other: Hispanic	12.75		
	Other: Non-Hispanic	10.50	9.39	11.61

**Figure 3:**
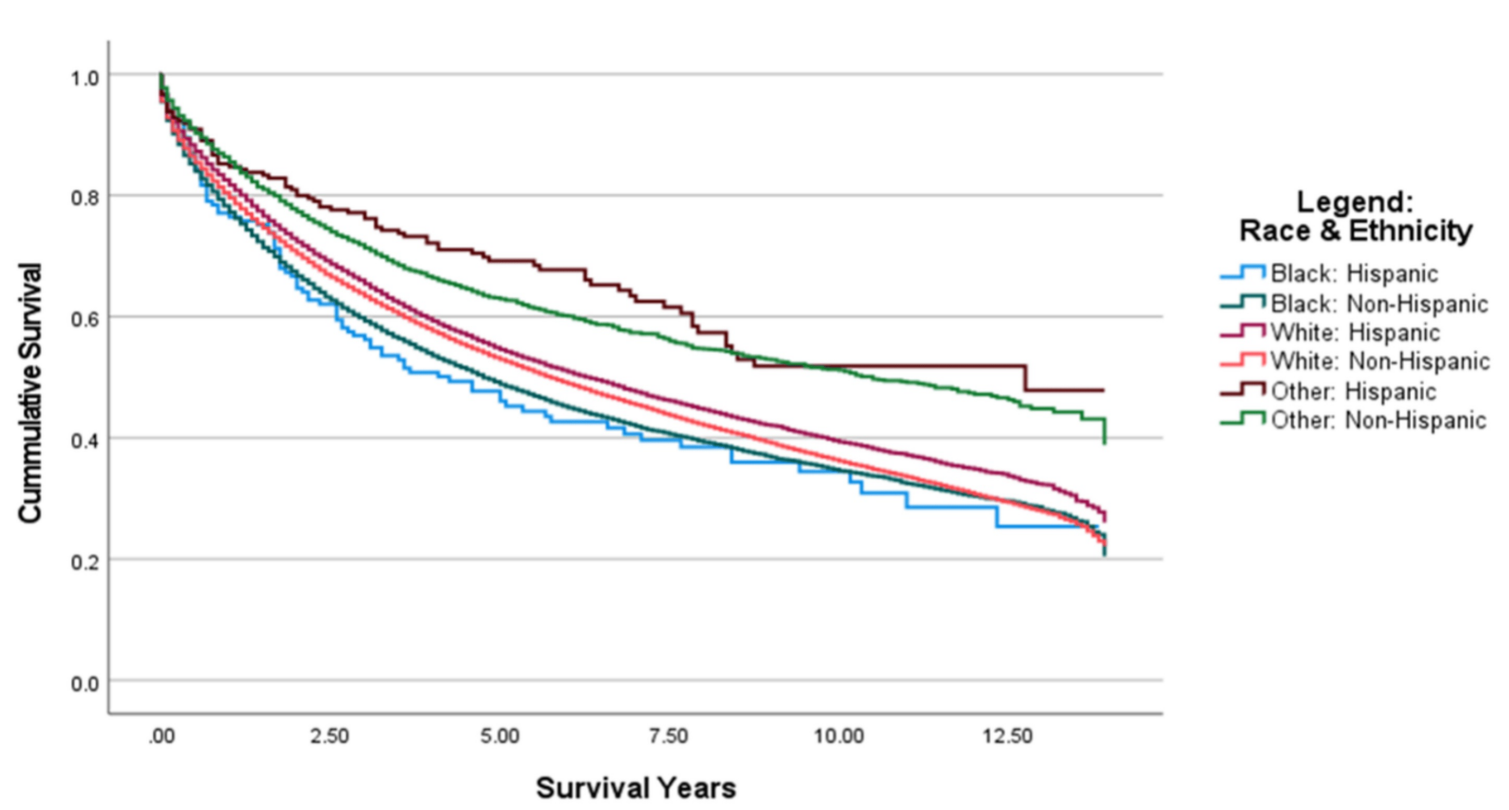
Cumulative Survival of Diagnosed CRC Patients by Race and Ethnicity From 2006–2016 CRC: Colorectal Cancer

**Figure 4:**
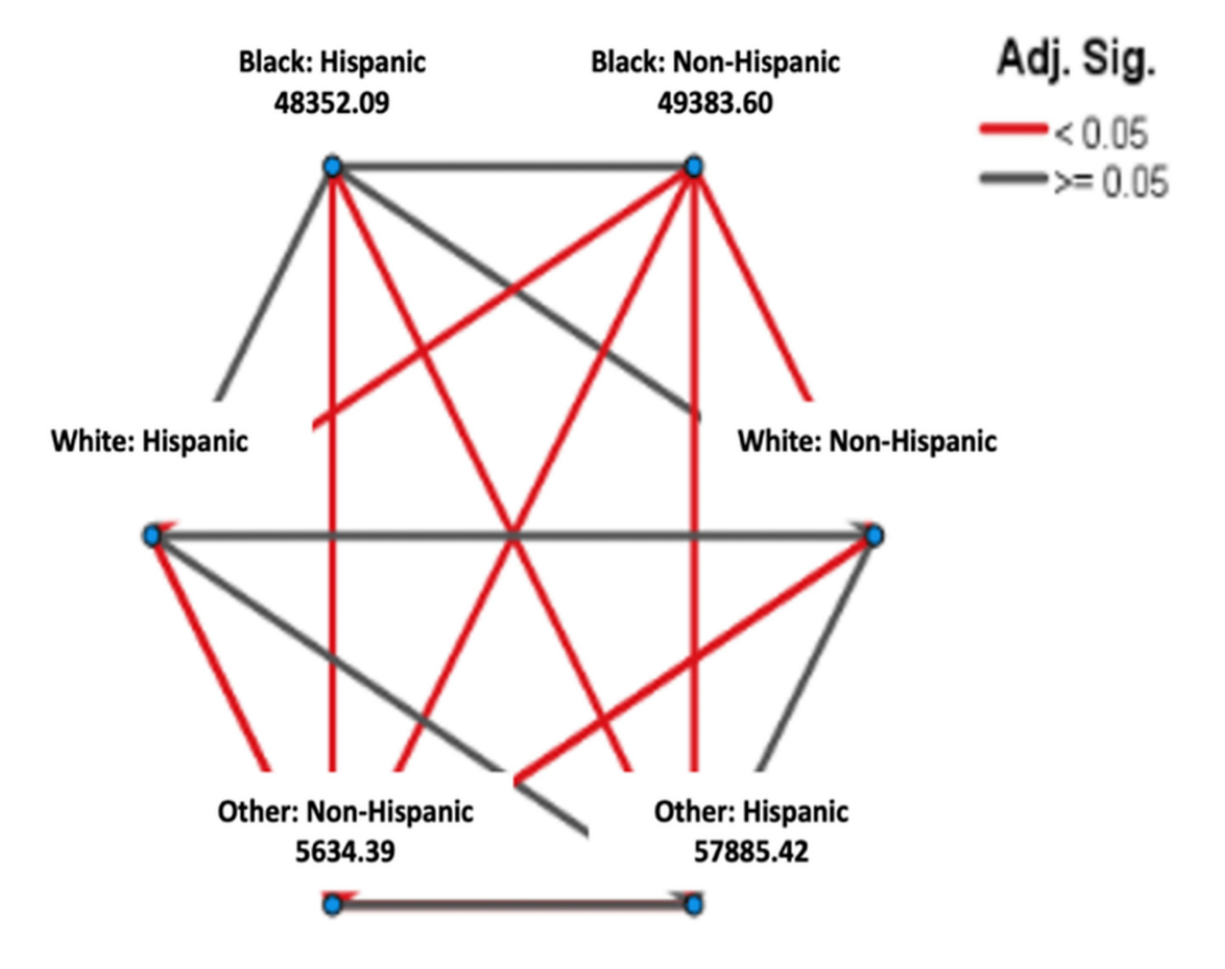
Pairwise Comparison of Survival Time Among Race/Ethnicity From 2006–2016

From 2006 to 2016, as shown in Figure 3, Black: Hispanic patients had the lowest median survival years (estimate = 4.250 years), followed by Black: Non-Hispanic patients (estimate = 4.750 years) and White: Non-Hispanic patients (estimate = 5.750 years) (Table 2). The variable, Other: Hispanic population, showed the highest median survival years (estimate = 12.750 years) (Table 2). The pairwise comparison of survival time among races and ethnicities between the years 2006 and 2016 showed that the Black: Non-Hispanic population was significantly different from all other races and ethnicities except Black: Hispanic (p < .001), as shown in Figure 4. The Black: Hispanic population was only significantly different from Other: Non-Hispanic population and Other: Hispanic (p<.001) population, as shown in Figure 4. The White: Hispanic population was significantly different from the Black: Non-Hispanic population and the Other: Non-Hispanic population (p<.001), as shown in Figure 4. The White: Non-Hispanic population was only significantly different from Other: Non-Hispanic population (p<0.001), as shown in Figure 4.

Intra-race and ethnicity comparison of survival time between 1995-2005 and 2006-2016 

Table 3 shows the results of the Kaplan-Meier Survival Functions for comparing the 10-year time periods of 1995-2005 and 2006-2016 within each race. There was no significant difference in the Black: Hispanic population's median survival time between 1995-2005 and 2006-2016 (Table 3). There was a significant difference in the Black: Non-Hispanic patients' median survival time between the two time periods (p=<0.001) (Table 3). There was a significant difference in White: Hispanic patients' median survival time between 1995-2005 and 2006-2016 (p=<0.001) (Table 3). There was a significant difference in the White: Non-Hispanic patients' median survival time between the two time periods (p=<0.001) (Table 3). Lastly, both Other: Hispanic patients and Other: Non-Hispanic patients showed no significant differences within their respective median survival times between 1995-2005 and 2006-2016 (Table 3). 

**Table 3:** Intra-race Median Survival Time Comparison From 1995–2016 With Pairwise Comparisons This Table Presents Median Survival Times and 95% Confidence Intervals (CIs) for Each Racial and Ethnic Group Across Two Time Periods (1995–2005 and 2006–2016). Pairwise Survival Comparisons Were Performed Using the Log-Rank Test. The Chi-Square (χ²) Statistic Is Reported for Each Comparison Where Applicable. A P-value < 0.001 Was Considered Statistically Significant and Is Shown in Bold. Note: A Dash (–) Indicates That the Comparison Was Not Statistically Tested Due to Limited Data or Non-significant Survival Differences.

Race/Ethnicity	Time Period	Median	95% C.I		Pairwise Comparisons	χ²
			Lower Bound	Upper Bound	p-value	
Black: Hispanic	1995-2005	5.00	3.40	6.60	-	-
	2006-2016	4.25	2.67	5.83		
Black: Non-Hispanic	1995-2005	3.58	3.41	3.76	<0.001	41.29
	2006-2016	4.75	4.56	4.94		
White: Hispanic	1995-2005	5.33	5.10	5.57	<0.001	35.71
	2006-2016	6.33	6.14	6.53		
White: Non-Hispanic	1995-2005	5.00	4.91	5.09	<0.001	45.02
	2006-2016	5.75	5.65	5.85		
Other: Hispanic	1995-2005	10.50	4.39	16.61	-	-
	2006-2016	12.75	-	-		
Other: Non-Hispanic	1995-2005	11.83	10.44	13.22	-	-
	2006-2016	10.50	9.39	11.61		

Determinants of race and ethnicity on age at diagnosis between 1995-2005 and 2006-2016 

Table 4 shows the intra-race comparison of mean age at diagnosis between 1995-2005 and 2006-2016. Black: Non-Hispanic, White: Hispanic, and White: Non-Hispanic patients showed significant differences between age at diagnosis when comparing the two time periods (p<0.001) (Table 4). 

**Table 4:** Games-Howell Analysis of Age at Diagnosis by Time Period Within Racial Groups Pairwise Comparisons of Mean Age at Colorectal Cancer (CRC) Diagnosis Across Racial and Ethnic Groups During 1995–2005 and 2006–2016, Using the Games-Howell Post-hoc Test Following Welch ANOVA. The Test Accounts for Unequal Variances and Sample Sizes. Mean Differences, Confidence Intervals (CIs), and P-values Are Reported. A P-value < 0.05 Was Considered Statistically Significant.

		1995-2005				2006-2016			
Race & Ethnicity	Comparison Race & Ethnicity	Mean Difference in Age at Diagnosis	Sig.	95% C.I.		Mean Difference in Age at Diagnosis	Sig.	95% C.I.	
				Lower Bound	Upper Bound			Lower Bound	Upper Bound
Black:Hispanic	Black: Non-Hispanic	-4.02	0.031	-7.82	-0.22	-0.57	0.995	-3.67	2.54
	White: Hispanic	-2.50	0.400	-6.30	1.29	-0.19	1.000	-3.29	2.91
	White: Non-Hispanic	-7.67	<0.001	-11.46	-3.89	-4.60	<0.001	-7.69	-1.50
	Other: Hispanic	2.57	0.764	-2.96	8.10	2.21	0.687	-2.12	6.54
	Other: Non-Hispanic	-0.45	0.999	-4.38	3.48	0.41	0.999	-2.76	3.58
Black: Non-Hispanic	White: Hispanic	1.52	<0.001	1.02	2.03	0.37	0.104	-0.04	0.79
	White: Non-Hispanic	-3.65	<0.001	-4.06	-3.25	-4.03	<0.001	-4.39	-3.67
	Other: Hispanic	6.59	<0.001	2.47	10.72	2.78	0.104	-0.31	5.87
	Other: Non-Hispanic	3.57	<0.001	2.43	4.72	0.97	0.007	0.17	1.78
White: Hispanic	White: Non-Hispanic	-5.17	<0.001	-5.54	-4.81	-4.40	<0.001	-4.71	-4.10
	Other: Hispanic	5.07	0.007	0.95	9.19	2.41	0.222	-0.68	5.49
	Other: Non-Hispanic	2.05	<0.001	0.92	3.18	0.60	0.242	-0.18	1.38
White: Non-Hispanic	Other: Hispanic	10.24	<0.001	6.13	14.35	6.81	<0.001	3.74	9.88
	Other: Non-Hispanic	7.22	<0.001	6.13	8.31	5.00	<0.001	4.25	5.76
Other: Hispanic	Other: Non-Hispanic	-3.02	0.314	-7.26	1.22	-1.81	0.571	-4.96	1.35

Years 1995-2005 

**Figure 5:**
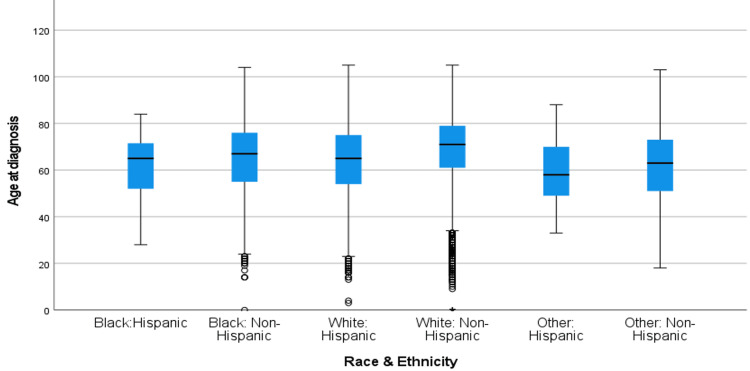
Age at diagnosis of CRC by Race/Ethnicity from 1995-2005

Figure 5 shows the box and whisker plot of age at diagnosis over the 1995-2005 time period across race and ethnicity. Table 4 shows the mean difference in age at diagnosis for all race and ethnicity variables. Table 4 shows the results of the post-hoc comparison of the Welch ANOVA function for the relationship between race and ethnicity and age at diagnosis during 1995-2005. Black: Hispanic patients showed an earlier age at diagnosis of CRC when compared to Black: Non-Hispanic patients (mean = -4.023, p < 0.032) and White: Non-Hispanic patients (mean = -7.67, p =< 0.001). Black: Non-Hispanic patients showed an earlier age at diagnosis than White: Non-Hispanic patients (mean = -3.65, p < 0.001) and showed a later age at diagnosis of CRC than all other races and ethnicities (p=<0.001). White: Hispanic patients showed an earlier age at diagnosis than White: Non-Hispanic patients (mean = -5.17, p=<0.001) but a later diagnosis than Other: Hispanic patients (mean = 5.07, p=<0.008) and Other: Non-Hispanic patients (mean = 2.05, p=<0.001). White: Non-Hispanic patients showed a later age at diagnosis than Other: Hispanic patients (mean = 10.2, p=<0.001) and Other: Non-Hispanic patients (mean = 7.22, p=<0.001). Lastly, Other: Hispanic patients showed no significant differences between Other: Non-Hispanic patients when looking at age at diagnosis of CRC (mean = 3.02, p=<0.05) as shown in Table 4. 

Years 2006-2016 

**Figure 6:**
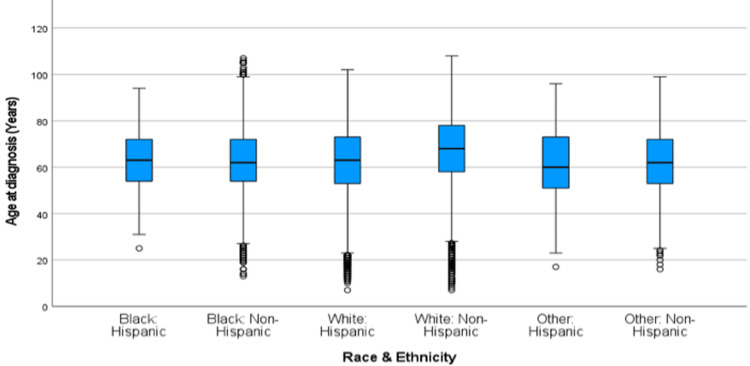
Age at diagnosis of CRC by Race/Ethnicity from 2006-2016

Figure 6 shows the box and whisker plot of age at diagnosis over the 2006-2016 time period across race and ethnicity. Table 4 shows the mean age at diagnosis for all race and ethnicity variables during the same time period. Table 4 shows the results of the post-hoc comparisons of the Welch ANOVA function for the relationship between race and ethnicity and age at diagnosis from 2005-2016. Black: Hispanic patients showed an earlier age at diagnosis of CRC when compared to White: Non-Hispanic patients (mean= -4.60, p=<0.001). Black: Non-Hispanic patients showed an earlier age at diagnosis of CRC than White: Non-Hispanic patients (mean= -4.03, p=<0.001) and showed a later age at diagnosis of CRC than Other: Non-Hispanic patients (mean = .974, p=<0.008). White: Hispanic patients showed an earlier age at diagnosis than White: Non-Hispanic patients (mean = -4.40, p=<0.001). White: Non-Hispanic patients showed a later age at diagnosis of CRC than all other races and ethnicities (p=<0.001). Lastly, Other: Hispanic patients showed an earlier age at diagnosis of CRC than White: Non-Hispanic patients (mean = -6.81, p=<0.001) as shown in Table 4. 

Determinant of sex on age at diagnosis between 1995-2005 and 2006-2016 

Years 1995-2005

**Figure 7:**
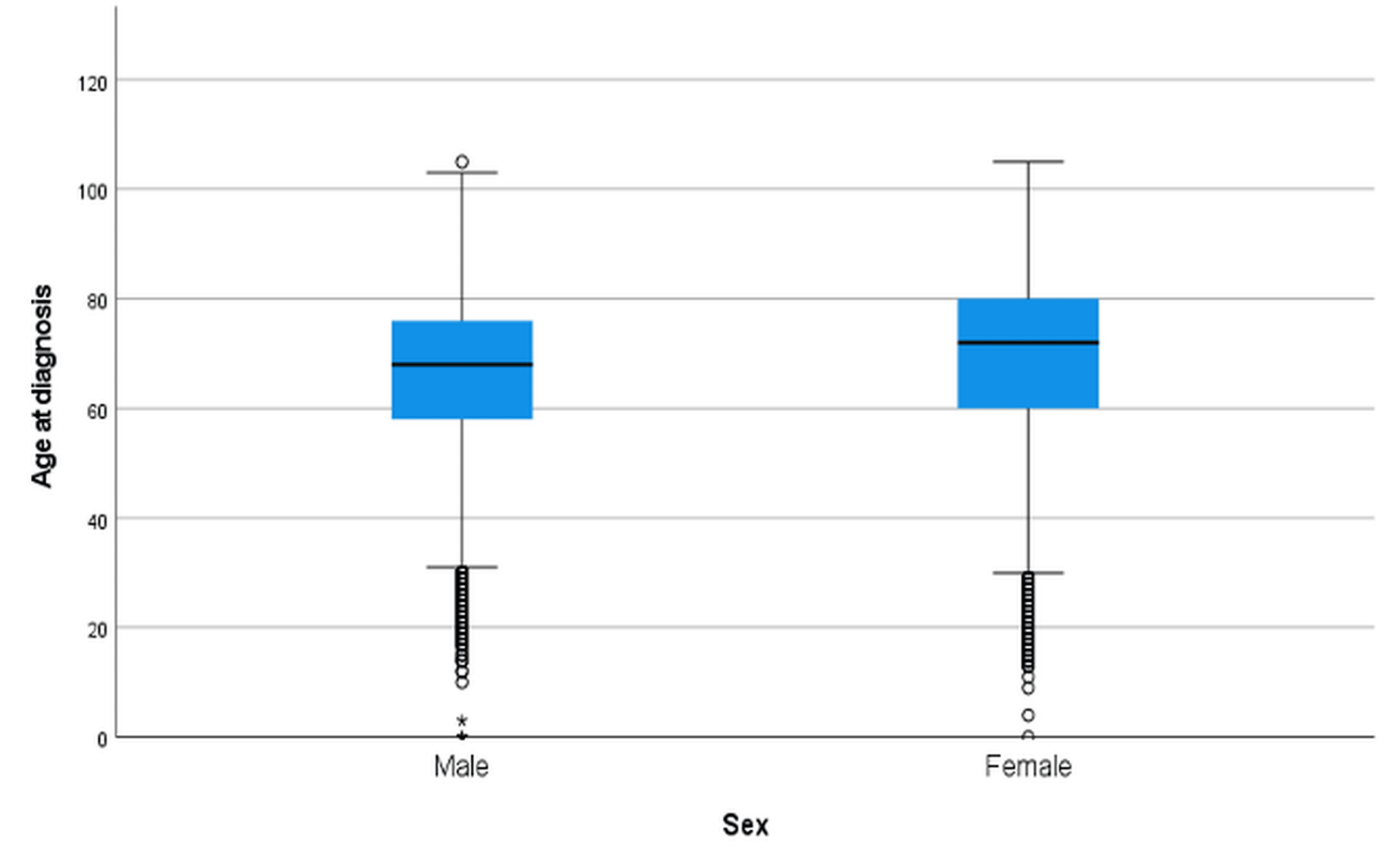
Age at diagnosis of CRC by sex from 1995-2005

Figure 7 demonstrates the age at diagnosis of CRC across sexes for the 1995-2005 period. Males (n = 50,377) had a mean age at diagnosis of 66.67 years, which was significantly lower than that of females (n = 45,772) with a mean age of 69.42 years (Table 5). As shown in Table 5, the Mann-Whitney U test revealed a statistically significant difference between the groups as shown on (Z = 34.520, p < 0.001).

**Table 5:** Age at diagnosis of CRC across sex: Results of the Mann-Whitney U test This table shows the results of the Mann-Whitney U test comparing mean age at colorectal cancer (CRC) diagnosis between males and females across two time periods (1995–2005 and 2006–2016). Z-scores and p-values are reported. A p-value < 0.05 was considered statistically significant. Statistically significant differences are indicated by p < 0.001.

Time Period	Variable	Total N	Mean Age at Diagnosis	Z-Score	p-value
1995-2005	Male	50377	66.67	34.52	< .001
	Female	45772	69.42		
2006-2016	Male	57722	64.69	21.57	< .001
	Female	47934	66.48		

Years 2006-2016

**Figure 8:**
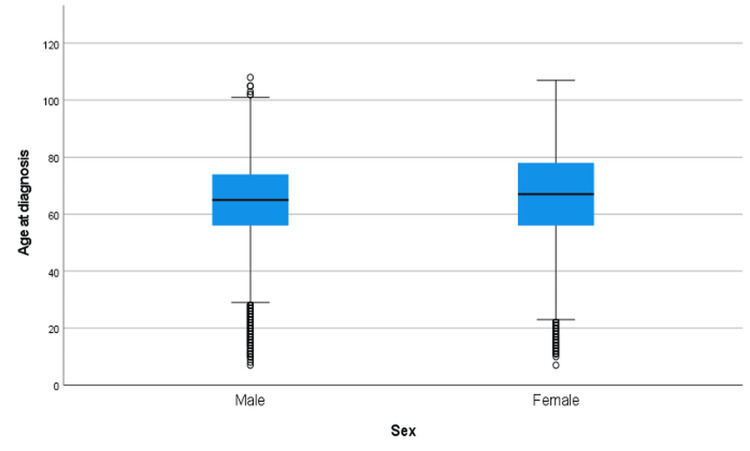
Age at diagnosis of CRC by sex from 2006-2016

Figure 8 illustrates the age at which CRC was diagnosed across sexes for the 2006-2016 period. Males (n = 57,722) had a mean age at diagnosis of 64.69 years, significantly lower than that of females (n = 47,934), whose mean age at diagnosis was 66.48 years. As shown in Table 5, the Mann-Whitney U test confirmed this difference was statistically significant (Z=21.57; p < 0.001).

Determinant of SES on age at diagnosis between 1995-2005 and 2006-2016

Years 1995-2005

**Figure 9:**
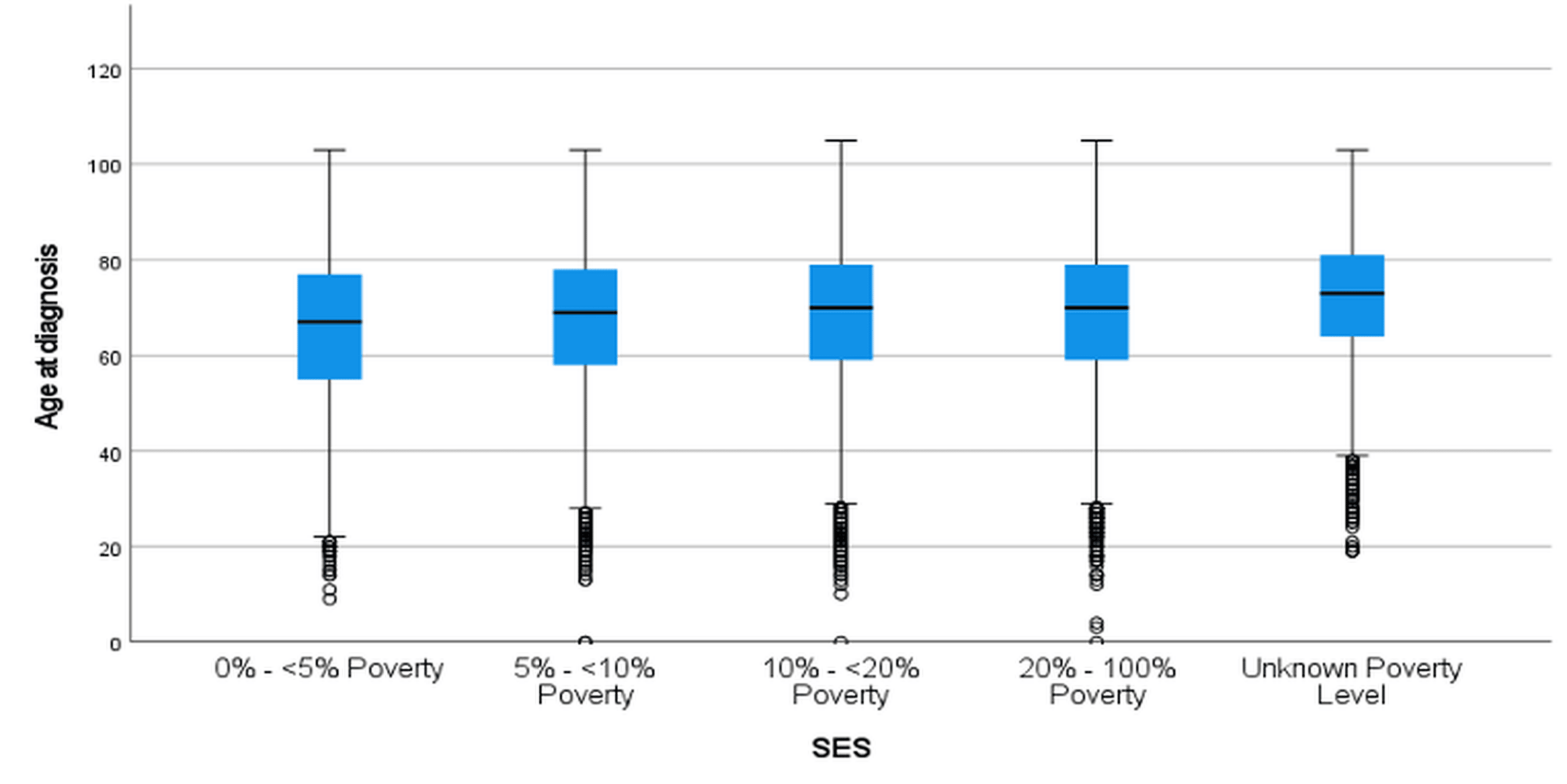
Age at Diagnosis of CRC by Socioeconomic Status From 1995–2005 CRC: Colorectal Cancer

Figure 9 illustrates the age at diagnosis of CRC stratified by SES for the 1995-2005 period. Across all SES categories, the median age at diagnosis remained relatively consistent. However, individuals in the "unknown poverty level" group exhibited slightly higher variability in age at diagnosis, as shown by the wider IQR. Comparisons between SES categories, as shown in Table 6, revealed that individuals in higher poverty levels (e.g., 20%-100% Poverty) were diagnosed at a younger age compared to those in lower poverty categories. The greatest differences in age at diagnosis were observed between the 0%-<5% poverty group and the unknown poverty level group (mean difference = -5.72, p < 0.001). Similarly, significant differences were noted between the 0%-<5% poverty group and higher poverty groups (e.g., 20%-100% poverty; mean difference = -2.53, p < 0.001). The Games-Howell post-hoc test demonstrated significant differences in age at diagnosis, particularly between the 0%-<5% poverty group and all other SES categories (p < 0.001).

**Table 6:** Games-Howell Analysis of Age at Diagnosis by Time Period Within SES This Table Presents Pairwise Comparisons of Mean Age at Colorectal Cancer (CRC) Diagnosis Across Socioeconomic Status (SES) Categories for Two Time Periods (1995–2005 and 2006–2016), Using the Games-Howell Post-hoc Test Following Welch ANOVA. This Method Accounts for Unequal Variances and Sample Sizes. Mean Differences, 95% Confidence Intervals (CIs), and P-values Are Reported. A P-value < 0.05 Was Considered Statistically Significant. Statistically Significant Differences Are Indicated By by p < 0.001.

		1995-2005				2006-2016			
Socioeconomic Status	Comparison SES	Mean Difference in Age at Diagnosis	Sig.	95% C.I.		Mean Difference in Age at Diagnosis	Sig.	95% C.I.	
				Lower Bound	Upper Bound			Lower Bound	Upper Bound
Affluent (0% - <5% Poverty)	Upper-Middle Income	-1.31	<0.001	-1.73	-0.89	-0.85	<0.001	-1.27	-0.43
	Lower-Middle Income	-2.51	<0.001	-2.89	-2.12	-1.53	<0.001	-1.91	-1.15
	High Poverty	-2.53	<0.001	-2.93	-2.13	-1.23	<0.001	-1.62	-0.85
	Unknown Poverty Level	-5.72	<0.001	-6.27	-5.17	-2.96	<0.001	-4.41	-1.51
Upper-Middle Income (5% - <10% Poverty)	Affluent	1.31	<0.001	0.89	1.73	0.85	<0.001	0.43	1.27
	Lower Middle-Income	-1.20	<0.001	-1.54	-0.85	-0.68	<0.001	-1.02	-0.34
	High Poverty	-1.22	<0.001	-1.57	-0.86	-0.39	0.018	-0.73	-0.04
	Unknown Poverty Level	-4.41	<0.001	-4.93	-3.89	-2.11	<0.001	-3.55	-0.67
Lower Middle Income (10% - <20% Poverty)	Affluent	2.51	<0.001	2.12	2.89	1.53	<0.001	1.15	1.91
	Upper-Middle Income	1.20	<0.001	0.85	1.54	0.68	<0.001	0.34	1.02
	High Poverty	-0.02	1.000	-0.34	0.30	0.30	0.030	0.01	0.58
	Unknown Poverty Level	-3.21	<0.001	-3.71	-2.72	-1.42	0.05	-2.85	0.00
High Poverty (20% - 100% Poverty)	Unknown Poverty Level	-3.19	<0.001	-3.70	-2.68	-1.72	0.01	-3.15	-0.29

Years 2006-2016

**Figure 10:**
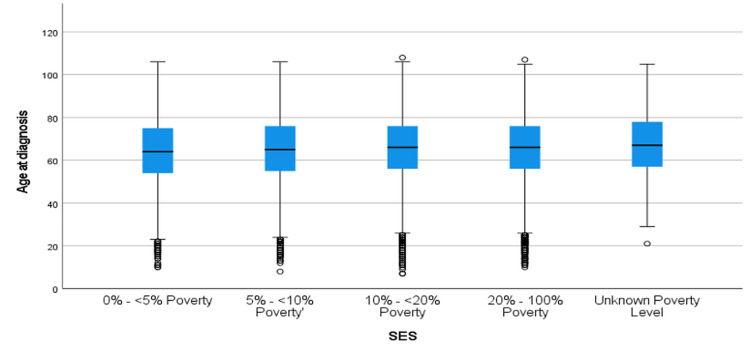
Age at Diagnosis of CRC by Socioeconomic Status From 2006–2016 CRC: Colorectal Cancer

Figure 10 shows the distribution of age at CRC diagnosis for the 2006-2016 period across SES categories. While the median age remained stable across SES groups, the overall trend mirrors that of the 1995-2005 period, with individuals in higher poverty levels diagnosed at younger ages. Notably, the variability in the "unknown poverty level" group persisted, and significant differences were observed in pairwise comparisons, as shown in Table 6. The largest differences observed were between the 0%-<5% poverty group and the unknown poverty level group (mean difference = -2.96, p < 0.001). Differences between the 0%-<5% poverty group and the 20%-100% poverty group were smaller but still statistically significant (mean difference = -1.23, p < 0.001). For instance, individuals in the 20%-100% poverty group were diagnosed at a significantly younger age than those in the 0%-<5% poverty group (p < 0.001).

## Discussion

This survival study aims to determine whether a significant difference exists in the time trend for individual races between ethnicities diagnosed with CRC and the impact of race, ethnicity, sex, and SES on age at diagnosis from the years 1995-2005 and 2006-2016. Our analysis revealed notable differences in CRC survival rates and age at diagnosis among different racial and ethnic groups over the two distinct time periods examined, from 1995-2005 and 2006-2016.

Comparison between race and ethnicity and survival time within a 10-year period from 1995-2005 and 2006-2016 

In the time period 1995-2005, Black: Non-Hispanic patients had the lowest median survival time when compared to the other races and ethnicities (Table 2). In comparison, Hispanic: Other patients had the highest median survival time (Table 2). Of interest, these figures differed in the 10-year period between 2006 and 2016. Black: Hispanic patients were found to have the lowest median survival time (Table 2), whereas Hispanic: Other patients remained the same with the highest median survival time (Table 2). These results indicate a persistent disparity in survival outcomes, with Black patients facing the lowest median survival time across both periods from 1995-2016. Similar studies have concluded that nationally, rates in CRC survival differ among races, with Black Americans showing the highest incidence and highest mortality among major U.S. racial groups [25]. 

From 2006-2016, Black: Hispanic and Black: Non-Hispanic patients exhibited some of the lowest median survival times among racial and ethnic groups (Figure 4). Although no statistically significant difference was observed between these two groups, both continued to experience poor survival outcomes, which aligns with prior studies highlighting disparities in CRC care. These disparities have been attributed to factors such as treatment delays and undertreatment among minority populations [19]. In contrast, individuals in the Hispanic: Other category consistently demonstrated the highest median survival time (Table 2). The Kaplan-Meier results emphasize the importance of considering racial and ethnic differences in CRC survival, with broader systemic and socioeconomic factors likely contributing to the observed disparities. It is important to interpret the survival estimates for the “Other” racial and ethnic categories with caution. These groups had substantially smaller sample sizes and wider confidence intervals compared to other populations, which may limit the reliability of their median survival estimates. Furthermore, the “Other” category likely encompasses a heterogeneous mix of racial and ethnic subgroups, reducing the specificity and interpretability of the findings. These limitations highlight the need for more granular and disaggregated data collection in future studies to better capture the nuances within these populations.

Intra-race and ethnicity comparison of survival time between 1995-2005 and 2006-2016 

Comparing intra-race and ethnicity survival time between both time periods illuminates disparities that may have occurred between the two 10-year periods. Analyzing the data in this manner lets us delve into changes that may have occurred due to better medical technology, screening, and treatment plans. There was no significant difference in the median survival time of Black: Hispanic patients between the time periods, showing a median estimate of 4.6 survival years. This continues the trend of Black American patients combating the social determinants of health. According to recent studies, patients with a greater number of social determinants of health were more likely to be female, Black, reside in the stroke belt, and have a higher comorbidity burden [26]. The significant difference in the Black: Non-Hispanic, White: Hispanic, and White: Non-Hispanic patients' median survival times between the time periods is intriguing. In the 1995-2005 period, the median survival years for Black: Non-Hispanic patients were 3.6 years, while the estimated survival years in the 2006-2016 period showed an estimate of 4.8 years. For White Hispanic patients, the median survival years in 1995-2005 was 5.33 years and 6.33 years in the 2006-2016 period. The White: Non-Hispanic patients had a median survival time of five years in 1995-2005 and 5.8 years in 2006-2016. Consistent with expectations, the median survival years for these three groups increased in the second time period. As advancements in medical technology, screening protocols, and treatment modalities evolve, it is reasonable to anticipate an improvement in median survival outcomes. However, it is interesting that not all races and ethnicities had improved survival times. Specifically, Black: Hispanic patients had no significant difference between the two time periods. The lack of improvement in median survival time underscores the need for targeted interventions to address the underlying factors contributing to disparities in healthcare access and outcomes. According to Musselwhite et al., screening rates vary considerably by race, ethnicity, and socioeconomic status in the United States. Rates are highest among White American patients (71.1%) and lowest among Hispanic American patients (56.1%). Black American patients (70.1%), American Indian/Native American patients (62.1%), and Asian American/Pacific Islander patients (64.8%) also have lower rates than White American patients [27]. Efforts to improve health equity must focus on addressing structural barriers to access, socioeconomic disparities, and social determinants of health that disproportionately affect minority populations.

Determinants of race and ethnicity on age at diagnosis between 1995-2016 

Overall, the mean age at diagnosis for all races and ethnicities decreased when comparing the two time periods. An intra-race analysis of age at diagnosis between the two decades showed interesting results (Table 4). Black: Non-Hispanic, White: Hispanic, and White: Non-Hispanic patients showed a statistically significant earlier age at diagnosis when comparing the two time periods (Table 4). The decreasing age at diagnosis for these races reflects trends seen in recent years, noting differences in incidence rates of early-onset CRC among Black patients compared to White and Hispanic patients [16]. 

The analysis of race and ethnicity on the age of diagnosis of CRC highlights some of the disparities disproportionately affecting ethnic minorities. The data demonstrate significant differences in the age at diagnosis of CRC among different racial and ethnic groups across both time periods. Interestingly, Hispanic ethnicity contributed considerably to an earlier onset of CRC compared to non-Hispanic counterparts. Factors such as SES, cultural beliefs, healthcare utilization, and access to preventative services could all play a role in influencing the timing of CRC onset among the Hispanic population [28]. From 1995 to 2005, Black: Hispanic patients were diagnosed with CRC at a younger age than Black: non-Hispanic and White: non-Hispanic patients. Similarly, from 2006 to 2016, Black: Hispanic patients were diagnosed at a younger age compared to White: non-Hispanic patients. Overall, Black: Hispanic patients consistently showed a younger age at diagnosis compared to White: non-Hispanic patients. This is reflective of previous literature stating that Hispanic and/or Black patients have poorer survival outcomes [18]. Building on the findings of this study and existing literature, the future of CRC screening may require more tailored approaches for ethnic minorities. Given the evidence of earlier diagnosis and poorer survival outcomes in these populations, future research should investigate whether screening should begin earlier for these groups. Tailored guidelines that account for the specific risks faced by ethnic minorities could potentially extend survival and delay disease onset, particularly when paired with targeted education at the time of screening. Before 2018, the American Cancer Society (ACS) recommended initiating CRC screening at age 50, with only the American College of Gastroenterology and the American Society for Gastrointestinal Endoscopy advising earlier screening for African Americans [29]. In 2018, the ACS revised its guidelines, lowering the recommended screening age to 45 for the general population [29]. However, no specific age adjustments were made for African American or Hispanic populations, despite their demonstrated increased risk. This underscores the need for future studies to evaluate the impact of earlier screening in high-risk ethnic groups and whether more personalized screening protocols could improve outcomes. 

Intriguingly, unlike the first period, the latter period showed no significant difference between Black: Hispanic patients' vs. Black: Non-Hispanic patients' age at diagnosis (Table 4). This could indicate two things: there is an improvement in access to quality care for Black: Hispanic patients, or there is a deterioration of care for Black: Non-Hispanic patients. This discrepancy warrants further investigation into potential systemic barriers or disparities in healthcare delivery. Furthermore, from 1995 to 2005, White: Non-Hispanic patients had a later diagnosis than White: Hispanic patients (Table 4). This difference identifies that ethnicity likely has an impact on the age at diagnosis when race is maintained as a constant. From 2006 to 2016, Black: Non-Hispanic patients had an earlier diagnosis than White: Non-Hispanic patients and a later diagnosis than Other: Hispanic patients (Table 4). This finding is consistent with previous literature stating Black patients have an increased risk of an earlier age at diagnosis than White patients, and that Other: Hispanic patients have the earliest diagnosis of all racial categories [18]. In both time periods, White: Hispanic and White: Non-Hispanic patients showed similar trends. White: Hispanic patients were diagnosed earlier than White: Non-Hispanic patients across both decades, while White: Non-Hispanic patients were consistently diagnosed later compared to other races and ethnicities (Table 4), as expected. 

Determinant of sex on age at diagnosis between 1995-2016 

Statistically significant differences in age at diagnosis between males and females were observed in both decades studied (Table 5). Across the two periods, males were consistently diagnosed at a younger age than females. From 1995 to 2005, the average age at diagnosis for males was nearly three years younger than for females. In the later decade, 2006-2016, this gap decreased, with males being diagnosed 1.8 years earlier than females.

The disparity in age at diagnosis likely reflects the interplay of environmental and genetic factors. Behavioral and lifestyle factors such as diet, physical activity, smoking, alcohol consumption, and adherence to traditional gender roles in the southern United States may further impact these trends. Sex-dependent lifestyle differences have been documented, with males typically consuming more meat, alcohol, and tobacco while eating fewer fruits and vegetables and leading more sedentary lives compared to females [30]. In Texas, these factors likely play an even larger role in the development of CRC, as the southern dietary pattern is characterized by high consumption of added fats, fried food, organ meats, processed meats, and sugar-sweetened beverages and is associated with increased risk of chronic diseases and carcinogenesis [31]. Additionally, the robust expansion of fast-food chains and processed food consumption also likely contributed to the earlier age at diagnosis for both sexes.

Biological influences, such as the effects of sex hormones, may contribute to differences in disease onset and progression as well. It has been established that estrogen plays a significant role in protecting women against CRC [32]. Meanwhile, testosterone has recently been reported to increase susceptibility to acquiring CRC in males [33]. However, notably, the gap in age at diagnosis between males and females narrowed by over a year between the two time periods. This reduction could be attributed to shifting societal norms, improved awareness of disease symptoms, and advancements in diagnostic practices.

Determinant of SES on age at diagnosis between 1995-2016 

Statistically significant differences between SES groups and age at diagnosis were observed across the two decades studied (Table 6). Higher SES groups were consistently diagnosed at a younger age compared to lower SES groups. From 1995 to 2005, the age gap between the lowest and highest SES groups was over 2.5 years. In the following decade, 2006-2016, this gap narrowed to 1.23 years. The halving of this disparity could be attributed to several factors, including increased awareness, improved screening practices, enhanced access to healthcare facilities, and greater insurance coverage.

Following pivotal trials published in the New England Journal of Medicine by Mandel et al. in 1993, which demonstrated the efficacy of fecal occult blood testing for CRC screening, a global consensus emerged that CRC screening should be offered to all individuals aged 50 and older at average risk [34, 35]. The gradual adoption of these recommendations likely contributed to the improved age at diagnosis among lower SES groups during the later decade. As new screening guidelines and technologies are introduced, these disparities are expected to continue decreasing.

Efforts to improve healthcare access have also played a key role in reducing SES-related disparities. For example, research presented at Digestive Disease Week 2023 highlighted interventions to boost CRC screening rates in medically underserved populations. These strategies included multicomponent interventions (e.g., patient education, provider reminders), patient navigation programs (navigators assisting patients in navigating the healthcare system), and mailed stool-based testing kits with provider outreach. Such measures have shown significant success in increasing screening rates among populations typically associated with lower SES [36]. Additionally, the Affordable Care Act (ACA), instituted in 2010, expanded affordable health insurance to millions of Americans and may have contributed to the improved age at diagnosis in lower SES groups [37]. Policies like the ACA, alongside continued efforts from lawmakers, healthcare providers, and public health initiatives, hold promise for further reducing SES-related disparities in CRC diagnosis and outcomes.

Further research is needed to elucidate additional determinants driving disparities in the timing of CRC onset among different racial and ethnic groups and to identify opportunities for targeted interventions. This analysis highlights the impact of being a minority of ethnic background on age at diagnosis of CRC. Longitudinal studies examining changes in healthcare utilization patterns, access to screening programs, and healthcare policies over time can provide valuable insights into the evolving landscape of CRC disparities. Additionally, qualitative research exploring patient perspectives and experiences with CRC screening and healthcare access can inform the development of culturally tailored interventions to improve early detection and outcomes for individuals at risk of CRC. 

This study has several limitations that should be acknowledged. The analysis did not account for key variables such as other comorbidities or treatment specifics, which are known to influence colorectal cancer outcomes. Additionally, the retrospective design limits causal inferences, does not neutralize unknown confounding factors, and broad racial and ethnic group categorizations may obscure important heterogeneity within populations. This study aims to highlight the current trends in CRC outcomes in Texas, and future studies will focus on neutralizing confounding factors. Changes in screening guidelines, healthcare policies, or treatment practices over the study period were also not explicitly analyzed and may impact the findings. 

## Conclusions

This study highlights significant and persistent disparities in colorectal cancer outcomes in Texas across racial, ethnic, and socioeconomic groups from 1995 to 2016. Our findings demonstrate that Black: Hispanic and Black: Non-Hispanic patients consistently had the lowest median survival times, with minimal improvements over time. Meanwhile, Hispanic patients, despite faring better than Black patients, still faced worse outcomes than their White: Non-Hispanic counterparts. The observed racial and ethnic differences in survival and age at diagnosis suggest systemic barriers to healthcare access, screening, and timely diagnosis may contribute to poorer outcomes. Additionally, the results indicate that sex and socioeconomic status significantly influence CRC diagnosis patterns, with males being diagnosed earlier than females and lower-income individuals experiencing earlier onset. While these findings highlight important associations, they do not establish causality.

Given the observational and retrospective nature of this study, we cannot rule out unmeasured confounding variables that may influence the observed disparities. Therefore, any policy recommendations should be considered hypothesis-generating and guided by future prospective studies that account for clinical variables, such as tumor stage, comorbidities, and treatment differences. Nonetheless, these results underscore the need for improved screening accessibility, earlier detection strategies, and equitable treatment approaches for historically underserved populations. Addressing disparities in CRC outcomes will require a multifaceted approach, including culturally sensitive prevention efforts, policy reform, and investment in community-based healthcare infrastructure to promote health equity.
